# Recent Advances in Designing High‐Capacity Anode Nanomaterials for Li‐Ion Batteries and Their Atomic‐Scale Storage Mechanism Studies

**DOI:** 10.1002/advs.201700902

**Published:** 2018-04-30

**Authors:** Qiuhong Cui, Yeteng Zhong, Lu Pan, Hongyun Zhang, Yijun Yang, Dequan Liu, Feng Teng, Yoshio Bando, Jiannian Yao, Xi Wang

**Affiliations:** ^1^ Key Laboratory of Luminescence and Optical Information Ministry of Education Department of Physics School of Science Beijing Jiaotong University Beijing 100044 P. R. China; ^2^ Department of Chemistry Stanford University Stanford CA 94305 USA; ^3^ School of Physical Science and Technology Lanzhou University Lanzhou 730000 P. R. China; ^4^ Tianjin Key Laboratory of Molecular Optoelectronic Sciences Department of Chemistry Tianjin University, and Collaborative Innovation Center of Chemical Science and Engineering (Tianjin) Tianjin 300072 P. R. China; ^5^ World Premier International Center for Materials Nanoarchitectonics (WPI‐MANA) National Institute for Materials Science (NIMS) Namiki 1‐1 Tsukuba 305‐0044 Japan; ^6^ Australian Institute for Innovative Materials (AIIM) University of Wollongong Squires Way North Wollongong NSW 2500 Australia; ^7^ Beijing National Laboratory for Molecular Sciences (BNLMS) Institute of Chemistry Chinese Academy of Sciences Beijing 100190 China

**Keywords:** atomic‐scale storage mechanisms, high‐capacity anodes, in situ transmission electron microscopy, Li‐ion batteries

## Abstract

Lithium‐ion batteries (LIBs) have been widely applied in portable electronics (laptops, mobile phones, etc.) as one of the most popular energy storage devices. Currently, much effort has been devoted to exploring alternative high‐capacity anode materials and thus potentially constructing high‐performance LIBs with higher energy/power density. Here, high‐capacity anode nanomaterials based on the diverse types of mechanisms, intercalation/deintercalation mechanism, alloying/dealloying reactions, conversion reaction, and Li metal reaction, are reviewed. Moreover, recent studies in atomic‐scale storage mechanism by utilizing advanced microscopic techniques, such as in situ high‐resolution transmission electron microscopy and other techniques (e.g., spherical aberration‐corrected scanning transmission electron microscopy, cryoelectron microscopy, and 3D imaging techniques), are highlighted. With the in‐depth understanding on the atomic‐scale ion storage/release mechanisms, more guidance is given to researchers for further design and optimization of anode nanomaterials. Finally, some possible challenges and promising future directions for enhancing LIBs' capacity are provided along with the authors personal viewpoints in this research field.

## 1 Introduction

Lithium ion batteries (LIBs) have attracted paramount interests in the past decades, because they are considered to be one type of important power sources applied in portable electronics and even electric vehicles.^1^, ^2^ With the increasing demands of power density, energy density, as well as excellent cycling stability for energy storage devices, more attention has been focused on designing advanced anode materials with high cycling performance, large energy and power densities, and low cost.^3^ Graphite is the most commonly used commercial anode material, it shows a low capacity of 372 mAh g^−1^, which restricts the applications in the large‐scale energy storage units.^4^ The basic reason is that graphite is a single‐electron‐controlled anode. Compared with graphite, it is testified that other types of carbon (e.g., N‐doped carbon‐/graphene‐based (NC/NG)) anode materials are believed to be multiple‐electron (ME) anodes (or hold numerous active sites for Li^+^ storage) and thus become competitive candidates with higher capacities larger than 372 mAh g^−1^ for LIBs. Meanwhile, the ME anodes usually include various element materials (such as Ge, Sn, Mn, Fe, Co, Ni, Li) and some transition metal oxides, which are explored as the promising anode materials for LIBs due to their high theoretical capacities.^5^ In addition, it is expected that various nanostructured electrode materials have been designed to realize the much short electron/Li^+^ transport path and thus high rate capability.^6^ As a result of the research on alternative anodes for LIBs, these materials' electrochemical properties have been enhanced and enriched very significantly during the past decade.

Most of the studies focusing on anodes for LIBs can be directed toward nanomaterials in the different mechanisms: intercalation/deintercalation mechanism, alloying/dealloying reactions, conversion reaction, and Li metal reaction.^7^ Different structures and compositions of anode nanomaterials have different processes to achieve high‐performance LIBs. Although masses of exciting progress have been achieved in this field, the prospective anode nanomaterials still cannot totally replace graphite in LIBs, because of unknown factors and indefinable mechanisms. Therefore, it is very urgent to find an efficient way to understand the mechanisms of electrochemical performance and degradation behavior of the anode in order to design reliable batteries with high‐performance. Recently, the state‐of‐art in situ techniques, especially in situ transmission electron microscopy (TEM), spherical aberration‐corrected scanning transmission electron microscopy (spherical aberration‐corrected STEM), cryoelectron microscopy (cryo‐EM), and 3D imaging techniques, are proved to be powerful and reliable ways to study properties of nanomaterials at atomic scale, particularly in the field of the energy storage.^8^ In this way, more in‐depth underlying mechanisms have been unveiled to guide to design and explore prospective anodes.

This review highlights the recent progress in the field of high capacity anodes in LIBs. Initially, we will introduce the kind of anode nanomaterials with different performances, which can be classified in groups according to different mechanisms, such as intercalation/deintercalation reactions, alloying/dealloying reactions, conversion reaction, and Li metal reaction. Various types of anode materials with different structures and corresponding performances will be concluded as classified respectively. Then, considerable attention will be focused on the techniques for atomic‐scale study of the storage mechanism in LIBs. For instance, the advanced techniques including in situ TEM, spherical aberration‐corrected STEM, cryo‐EM, and 3D imaging techniques enable to acquire the direct observation of surface energy storage, volume variations, and strain changes so that we can unveil the ion storage/release mechanisms in atomic‐scale. Finally, based on the recent studies, there are more perspectives on what possible obstacles the anode materials will encounter and how these techniques can further enhance the electrochemical performance on the directions toward the future research in this area.

## 2 High‐Capacity Anode Nanomaterials for Li‐Ion Batteries

Carbon materials, typically graphite (theoretical capacity is 372 mAh g^−1^), are the most commonly used anodes materials in LIBs. However, the small (0002) interlayer distance of 0.34 nm and low capacity are the major limitations for graphite's application in high energy density battery. Recently, various advanced nanomaterials are mushrooming to substitute traditional anode materials, devoting great efforts to the next‐generation LIBs with high power and energy density. The energy density and capacity equations are as follows:

(1) $$\mathrm{ED}\;=\;nFE^{0}/\Sigma M_{i}$$

(2) $$C\;=\;\left(F\;\times \;n\right)/\left(3.6\;\times \;M\right)$$

Where ED represents mass density (Wh kg^−1^) or volumetric density (Wh L^−1^); *n* represents electron transport numbers; *E* ^0^ is potential; Σ*M*_i_ is total mass), it is clear to demonstrate that the performance of anodes in LIBs is related to electron transfer behaviors and the numbers of electron transport. Hence, all the high‐capacity anodes mentioned in this review are the ME materials. Compared with graphite, two sides of graphene layer can hold double times of Li‐ions forming LiC_3_ instead of LiC_6_ and thus perform higher capacity of 744 mAh g^−1^ (two times than that of graphite). Because of the high electric conductivity, good flexibility, and high chemical stability, graphene is believed as one of the most promising carbon matrices in the application of LIBs.

Recently, tremendous advanced anodes were designed following these properties: first, there are some elements or compounds with low atomic or formula weights in these anode materials. Second, their porous structure and low density are helpful to accommodate fairly large amounts of Li per formula unit. Third, they own cycling stability and reversible volumetric (mAh cm^−3^) capacities and other merits serving as excellent anodes. For example, some new anode materials such as Si (4200 mAh g^−1^), Sn (994 mAh g^−1^), and SnO_2_ (782 mAh g^−1^) have been widely investigated for their specific capacity. Furthermore, by utilizing progressive nanotechnology, researchers have fabricated a variety of anode materials with unique nanostructures, such as nanowires, nanotubes, and carbonaceous support materials for attractive features and properties. Based on the storage mechanism of Li ion, most of those prospective anodes can be classified in following types: intercalation/deintercalation, alloying/dealloying, conversion reaction, and Li metal reaction. In the light of the above‐mentioned classification, we summarized recent progress in developing and synthesizing anodes nanomaterials and reported their excellent electrochemical performances in LIBs in each section, respectively.

### 2.1 Intercalation/Deintercalation Mechanism

**Figure 1:**
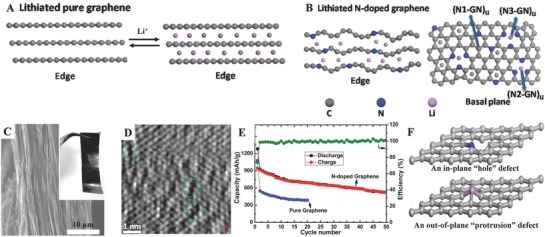
Illustrations of A) G and B) GN nanosheets for Li‐insertion. C) SEM image of the cross‐section of a piece of GN thin film; inset is a photograph showing the flexibility of a GN paper. D) HRTEM images of surface structural evolution of lithiated GN. E) Cycle performance and Coulombic efficiency.^19^ F) The side views of two optimized pnictogen–graphene monovacancy systems: an in‐plane “hole” defect produced by the pyridinic nitrogen‐doping (blue); an out‐of‐plane “protrusion” defect through the phosphorus substitution (pink).^21^ Panels (A–E) Reproduced with permission.^19^ Copyright 2014, American Chemical Society. Panel (F) Reproduced with permission.^21^ Copyright 2017, Royal Society of Chemistry.

Li‐ions can reversibly intercalate and deintercalate into the lattice of layer anode materials without destroying the crystal structure, which belongs to the intercalation/deintercalation mechanism. Remarkably, Li‐ions transport in carbon‐based anode material (e.g., graphite) follows this mechanism (**Figure** **1**A), but their storage capacities are seriously restricted by the narrow lattice distance. Hence, many new methods are put forward to release the restriction by carbonaceous materials' inherited layer structures.^9^, ^10^, ^11^ One of the strategies is to doping elements in carbon such as N and P or to modify the functional groups on the surface, so that the available active sites can be increased and the electronic properties can also be effectively modulated.^12^, ^13^ Actually, these doped materials have the intrinsically superior electrical conductivity that accelerates electrons' transport, high surface area, open and flexible porous structures available for numerous lithium storage sites, high Li^+^ diffusivity, and short Li^+^ diffusion distances.^13^, ^14^, ^15^, ^16^, ^17^, ^18^

For example, Wang et al. created a binder‐free N‐doped graphene (GN) paper anode, which devotes to exhibiting high capacity and ultrafast lithium storage property.^19^ Compared with Li storage process in pure graphene (G) nanosheet, the GN nanosheets exhibit obvious different storage mechanisms, as illustrated in Figure 1B. Clearly, GN owns larger average (0002) distance, as well as many holes and points defects, resulting in more active energy storage sites and therefore both improved rate performance and capacity. There are three kinds of N‐substituted configurations (N1‐GN, N2‐GN, and N3‐GN) on lithium adsorption in GN (Figure 1B). According to density functional theory calculation, each of N‐substituted configuration presents much higher capacities 1087, 1198, and 1262 mAh g^−1^ comparing with pure graphene (744 mAh g^−1^), respectively, because the reversible insertion/deinsertion of Li ions is improved by increasing number of active binding sites and Li‐insertion sites in GN are totally different from cases in G. As shown in Figure 1C, the as‐prepared GN nanosheets look like a piece of flexible paper with a uniform cross‐section and a disordered structure, exhibiting these sheets did not restack back to graphite. The high‐resolution transmission electron microscopy (HRTEM) image of the GN surface (Figure 1D) illustrates that more atom‐level N substitution hole defects or disordered sites appear on the surface. It suggests that the nitrogen doping can, to some extent, facilitate the Li‐insertion. During the first cycle, GN electrodes exhibit ultrahigh discharge capacity of 1284 mAh g^−1^, which is much higher than 1080 mAh g^−1^ of the pristine G (Figure 1E).

N‐doping induced surface defects in as‐fabricated graphene layers but attributed the success to make great progress in their electrochemical properties. Inspired by this, Ajayan and co‐workers reported that the GN layers are obtained through controlling fabricated method, which possess remarkable features of high reversible discharge capacity in the application for LIBs.^16^ Wang and co‐workers have designed a 3D branch‐rich architecture foam constructed by a tubular N‐doped graphitic network, which is ascribed to making dramatical enhancement in Li^+^ storage capacity and rate performance.^20^ Because of high surface area mountains in nature, it can be predicted that introducing blistering in graphene, instead of making “holes,” would be another effective way to adsorb/insert more Li^+^ and to further improve the capacity of LIBs (Figure 1F). Researchers have selected P as dopants to produce the out‐of‐plane protrusions in graphene. The formation of these protrusions is primarily driven by the rehybridization of p_z_ states of the vacant C sites toward overall stabilization of the pnictogen–graphene system, which leads to a larger charge transfer between the substituted heteroatoms and C atoms.^21^

Besides the doping modification of carbon‐based anodes, another strategy is to synthesize various nanostructures because they can shorten the diffusion pathways and offer large electrode/electrolyte interfaces for the charge‐transfer reaction and thus show the better electrochemical performance, such as carbon nanotubes,^22^ nanofibers,^23^ nanobeads,^24^ hollow nanospheres,^25^ porous carbon,^26^ and their hybrids.^27^, ^28^, ^29^ As shown in **Table** **1**, some recent carbon‐based anode nanomaterials and their corresponding performance in LIBs have been compared and summarized. Because of the effective ion transport channels and enriched active sites for Li, those anodes exhibit high capacity, rate capability, and stable cyclability, which would inspire new strategies toward creation of novel and promising anodes for LIBs.

**Table 1:** The designed recent carbon‐based anodes and their LIB properties

Carbon‐based anodes	1st and 2nd discharge capacity [mAh g^−1^]	Capacity after (*n*) cycles [mAh g^−1^]	Current density [mA g^−1^]	Ref.
Porous graphene networks	≈1000	850	≈370	209
Hollow carbon‐nanotube@carbon‐nanofiber	1530	1150	100	210
2D mesoporous carbon nanosheets	1040	833	100	211
N‐doped porous carbon capsules@carbon nanotubes	1235	1337	500	29
Mesoporous N‐rich carbons	1780	1365	100	212
Amorphous carbon nanotubes@hollow graphitic carbon nanospheres	1597	969	50	213
N‐doped multiwall carbon nanotubes	3500	350	200	12
Hierarchical porous N‐doped carbon nanosheets	1991	1913	100	214
N‐doped porous carbon nanofiber webs	1363	1280	100	213
Branched N‐doped graphitic tubular foam	≈1049	1049	500	20

Similar to carbon‐based anodes, some transition metal oxides, transition metal dichalcogenides (TMDs), and other compounds with a 2D layer structure or 3D network structure are applied to successfully explore the process of Li^+^ reversibly intercalating into the lattice. In order to start the above reaction involving Li^+^ occurs some required conditions are up to the standard in these materials. First, a transition metal or rare earth metal is required to be contained in the host compound; consequently it can exhibit one or more stable valence states. Second, the crystal lattice in the compound should be large enough to permit Li‐ions intercalation/deintercalation. For example, TMDs (MX_2_; M = Mo, Ti, V, and W, X = S or Se) share the similar feature of layered structure as graphite, but strong covalent bonds within layers and weak van der Waals forces between layers coexist in MX_2_, which provide ideal space for Li^+^ intercalation. To further facilitate Li^+^ intercalation/deintercalation, optimized nanostructures have been designed due to their inherent advantages, such as high surface area and short diffusion path for Li‐ion transport. Moreover, it is commonly accepted that fabricating nanocomposites enable to increase the conductivity of the whole electrode materials especially accompanied with carbonaceous materials and MX_2_. Take the MoS_2_ for an example to demonstrate the reaction of MX_2_.^30^ Within the voltages from 3.0 to 1.1 V, intercalation reaction occurs: MoS_2_ + *x*Li^+^ + *x*e^−^ → Li*_x_*MoS_2_ (≈1.1 V vs Li/Li^+^, 0 ≤ *x* ≤ 1),^31^ giving rise to 167 mAh g^−1^. The intercalation of Li^+^ would induce the lattice disruption of 2H‐Li*_x_*MoS_2_, which leads to the phase conversion of MoS_2_ and transformation of the MoS_6_ unit from trigonal (2H‐Li*_x_*MoS_2_) to octahedral (1T‐Li*_x_*MoS_2_) prisms.^32^, ^33^ While, when the voltage is lower than 1.1 V, the conversion reaction: Li*_x_*MoS_2_ + (4 – *x*) Li^+^ + (4 – *x*) e^−^ → Mo + 2Li_2_S (≈0.6 V vs Li/Li^+^) predominates and the theoretical capacity derived from both the intercalation and conversion reactions is 669 mAh g^−1^.^34^

**Figure 2:**
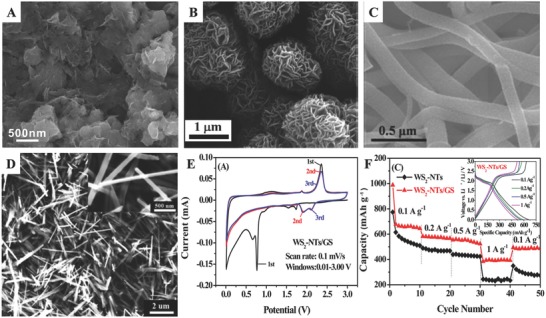
The SEM images of different nanostructures of TMDs‐based anodes: A) layered MoS_2_/graphene composites; Reproduced with permission.^35^ Copyright 2011, American Chemical Society. B) graphene/MoS_2_ nanoflake arrays; Reproduced with permission.^36^ Copyright 2013, Wiley. C) MoS_2_@G; Reproduced with permission.^30^ Copyright 2014, Royal Society of Chemistry and D) WS_2_–NTs/GS. Panels (D–F) Reproduced with permission.^37^ Copyright 2017, American Chemical Society. E) Cyclic voltammetry of the WS_2_–NTs/GS hybrid anode. F) Rate capabilities; inset: discharge/charge voltage profiles.

Chang and Chen fabricated layered MoS_2_/graphene composites with high reversible capacity and long‐term stability,^35^ as shown in **Figure** **2**A. Gao and co‐workers prepared a new 3D architecture consisted of graphene/MoS_2_ nanoflake arrays^36^ (Figure 2B). During charging and discharging, this 3D architecture can keep stable in several cycles and the porous characteristics can lead to form a large electrode/electrolyte interfacial area, resulting in high current density of 8000 mA g^−1^ and high rate performance in a similar vein. Furthermore, an optimized contact mode between MoS_2_ and graphene was synthesized by Yang et al.,^30^ as shown in Figure 2C, where graphene wraps thin MoS_2_ nanosheets building MoS_2_@G nanocable structure. The distinct MoS_2_@G nanocable structure in conducive to realize specific capacity up to 1150 mAh g^−1^ at 0.5 A g^−1^ and an impressive rate capability with a capacity of 700 mAh g^−1^ even at a current density of as high as 10 A g^−1^.

Amine group reported a simple one‐pot hydrothermal reaction for constructing novel 3D WS_2_ nanotubes/graphene (WS_2_‐NTs/GS) hybrid with unique sandwich‐type architecture^37^ (Figure 2D). The graphene‐based hierarchical architecture plays vital roles in achieving fast electron/ion transfer, leading to good electrochemical performance. Corresponding electrochemical characterizations were analyzed to investigate the anode performance of the WS_2_‐NTs/GS hybrid. The cyclic voltammograms in Figure 2E indicate the reversibility of lithiation and delithiation in cycles. As shown in Figure 2F inset, the charge/discharge profiles of the WS_2_‐NTs/GS hybrid anode demonstrate a high capacity retention upon cycling and the unchanged pattern of discharge and charge plateaus. The characterization of rate capabilities in Figure 2F shows the good rate performance and rate tolerance of WS_2_‐NTs/GS hybrid anode.

Another alternative to carbon materials is the “zero‐strain” spinel lithium titanate oxide (Li_4_Ti_5_O_12_),^38^ because of its negligible volume change (only 0.2–0.3%), a flat charge/discharge plateau (around 1.55 V vs Li/Li^+^), excellent safety characteristics (suppressed formation of solid electrolyte‐interphase (SEI) layer and avoided growth of lithium dendrites), and cycling stability.^39^, ^40^, ^41^ However, there are still a number of challenges remaining for Li_4_Ti_5_O_12_ battery applications, such as relatively low electronic conductivity, large polarization losses, and low ionic diffusion rate. Currently, extensively research efforts have been devoted to improving the performance of Li_4_Ti_5_O_12_ and some important progresses have been made.^42^, ^43^, ^44^

One approach is to enhance the electronic conductivity of spinel Li_4_Ti_5_O_12_ by surface treatments.^45^, ^46^ The other approach for enhancement of the Li ion diffusion is by downsizing the spinel Li_4_Ti_5_O_12_ to the nanoscale. For example, nano Li_4_Ti_5_O_12_ with particle sizes between 20 and 50 nm have been fabricated through simple combustion method in short time period.^47^ During the first discharge Li_4_Ti_5_O_12_ takes about 2.92 Li atoms corresponding to a capacity of 170 mAh g^−1^ that matches well with the expected theoretical capacity for Li_4_Ti_5_O_12_. While stable capacities of 140 and 70 mAh g^−1^ were observed at higher charg/discharge rates of 10 and 100 C, respectively. The electrical conductivity can also be enhanced by doping Li_4_Ti_5_O_12_. Shen et al. have grown Li_4_Ti_5_O_12_ nanowire directly on titanium foil and the conductivity of Li_4_Ti_5_O_12_ nanowires is improved by introducing Ti^+3^ ions through hydrogenation.^48^ Furthermore, a recent study on a carbon‐coated Li_4_Ti_5_O_12_ nanoporous microsphere showed a remarkable improvement in rate capability of 126 mAh g^−1^ at 20 C.^49^

### 2.2 Alloying/Dealloying Reaction

Getting involved with Li cycling in LIBs, some metallic and semi‐metallic elements can form alloys with Li metal, such as groups IV (Si, Sn, Ge, Pb, P, As, Sb, and Bi), and also some other metal elements, such as Al, Au, In, Ga, Zn, Cd, Ag, and Mg,^50^, ^51^, ^52^ which exhibit distinct physical properties that different from individual Li. These alloyed anodes are well known for high specific capacities, e.g., Si, Ge, Sn, etc. with capacity values of 4200, 1600, 999 mAh g^−1^, respectively, through an alloying product of Li_4.4_M (M = Si, Ge, and Sn).^53^, ^54^, ^55^ Because of the smaller atomic mass, Si elements usually exhibit larger capacity than other alloying materials. However, during Li alloying/dealloying reactions, those alloy‐based anode materials suffer from several critical challenges, such as slow lithium reaction kinetics, large volume changes, and poor intrinsic conductivities,^56^ which is the major factor for capacity fading in the long‐term cycle. To buffer such huge volume changes during Li alloying/dealloying reactions, approaches for designing special nanostructures of different dimensionalities like nanoparticles, nanowires,^57^ nanotubes,^58^ as well as for nanoporous structures^59^, ^60^ have been reported. Moreover, it is a good attempt to integrate the electrode material with a carbonaceous matrix^61^, ^62^, ^63^, ^64^ such as graphene, amorphous carbon, mesoporous carbon, carbon nanotubes, electrospun carbon fibers, hollow carbon nanotubes, and Si–C pomegranate structures for the high conductivity for electrons and stable structure in cycling. This section focuses on the use of elecemts (Si, Ge, Sn, Sb), since they provide high theoretical capacities of more than 900 mAh g^−1^ and they are relatively abundant.

**Figure 3:**
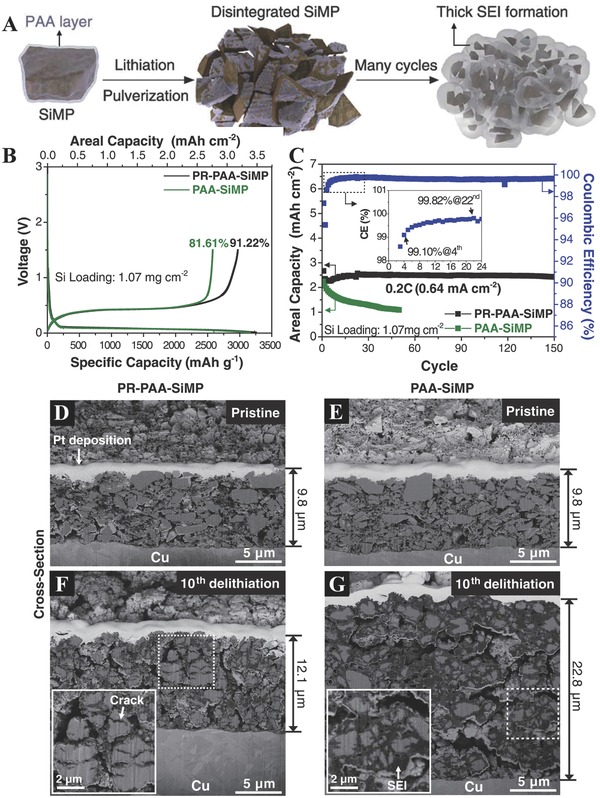
A) Schematic illustration of the pulverization of the PAA–SiMP electrode during cycling and its consequent SEI layer growth. B) The initial charge–discharge profiles of the PR‐PAA–SiMP and PAA–SiMP electrodes when measured at 0.033 C (100 mA g^−1^). C) Discharging capacity retentions of both electrodes when measured at 0.2 C (600 mA g^−1^). Cross‐sectional images of the D) PR‐PAA–SiMP and E) PAA–SiMP electrodes before cycling. Cross‐sectional images of the F) PR‐PAA–SiMP and G) PAA–SiMP electrodes after the 10th delithiation. Reproduced with permission.^72^ Copyright 2017, Science.

Silicon (Si), as one of the most important alloying elements, has attracted great interests as a promising anode for LIBs because of their high capacity (4200 mAh g^−1^) in the equilibrium Li–Si alloy phase.^65^ The electrochemical lithiation of Si electrodes has been deeply investigated by many groups. It has been clarified that the high specific capacity value is due to the formation of intermetallic Li*_x_*Si*_y_* binary compounds such as Li_12_Si_7_, Li_7_Si_3_, Li_13_Si, Li_4.4_Si. However, the use of silicon still remains challenging due to the large volume expansion (>400%) during the lithiation–delithiation process (as shown below) and formation of the SEI layer at low potential (**Figure** **3**A). Moreover, the formation of Si compounds at the solid electrolyte interface inhibits the alloy/dealloy process

(3) $$\begin{array}{c} \mathrm{lithiation}:\;x-\mathrm{Si}\begin{array}{c} \mathrm{Li}\\ \to \end{array}a-{\mathrm{Li}}_{y}\mathrm{Si}\begin{array}{c} \mathrm{Li}\\ \to \end{array}a-{\mathrm{Li}}_{x}\mathrm{Si}\begin{array}{c} \mathrm{Li}\\ \to \end{array}x-{\mathrm{Li}}_{1.5}{\mathrm{Si}}_{4}\\ \mathrm{delithiation}:\;x-{\mathrm{Li}}_{1.5}{\mathrm{Si}}_{4}\begin{array}{c} -\mathrm{Li}\\ \to \end{array}a-{\mathrm{Li}}_{z}\mathrm{Si}\begin{array}{c} -\mathrm{Li}\\ \to \end{array}a-\mathrm{Si}\;\;\;\;\;\;\;\;\;\;\;\;\\ \mathrm{Subsequent}\;\mathrm{cycling}:\;a-\mathrm{Si}\begin{array}{c} -\mathrm{Li}\\ \to \end{array}a-{\mathrm{Li}}_{z}\mathrm{Si}\begin{array}{c} -\mathrm{Li}\\ \to \end{array}x-{\mathrm{Li}}_{1.5}{\mathrm{Si}}_{4} \end{array}$$

“*x*” refers to a crystalline phase and “*a*” indicates an amorphous phase.

To overcome these issues, various high‐performance polymer binders have been developed to improve the cycle lives of Si anodes,^66^, ^67^, ^68^, ^69^ even if 3D polymer networks have been applied to achieve longterm cycle life.^70^, ^71^ Those binders usually have optimizing results in the Si nanoparticle anodes, while the positive effects of binders in Si microparticle (SiMP) anodes remain challenging. Choi developed a binder for SiMP anodes in which a small amount of ring‐slide polyrotaxane (PR) is covalently integrated with a conventional linear binder, polyacrylic acid (PAA).^72^ The electrode based on PR‐PAA showed a higher reversible capacity of 2971 mAh g^−1^ in the precycling at 0.033 C (100 mA g^−1^) compared with that of the PAA–SiMP electrode (2579 mAh g^−1^) (Figure 3B). This binder combination keeps even pulverized silicon particles coalesced without disintegration, enabling stable cycle life for silicon microparticle anodes at commercial‐level areal capacities. As shown in Figure 3C, the PR‐PAA–SiMP with an initial areal capacity of 2.67 mAh cm^−2^ preserved 2.43 mAh cm^−2^ after 150 cycles at 0.2 C (0.64 mA cm^−2^). The scanning electron microscopy (SEM) images in Figure 3D–G show the critical role of PR‐PAA on the electrochemical performance. After 10th delithiation, the change of thickness of the PR‐PAA–SiMP is smaller than that of PAA–SiMP electrodes, reflecting the enhanced stability of the SEI layer of the PR‐PAA–SiMP.

Besides the improvment of binders in LIBs, various Si nanomaterials and modified nanostructured Si materials such as Si nanowires,^57^, ^73^, ^74^ Si particles, Si hollow spheres, Si porous nanostructures, and Si@C yolk–shell structures,^60^, ^75^, ^76^, ^77^ have also been constructed as LIBs anode materials, which prove their excellent rate performance and cycling stability. For example, Mulder and co‐workers have synthesized the binder/carbon‐free Si nanopartical anode, which exhibits high specific areal capacity as well as cycling stability.^20^ The Si nanopartical anode shows sufficient electronic conductivity facilitated by the lithiation of Si throughout the solid. The difference in Si nanopartical size results in different electrode performance. In the nanosize range, the smaller particle size usually performs better than the larger size.^78^ Different types of carbon matrixes have been developed to embed the Si particles, which provided efficient electron transportation routes and space for volume expansion accommodation.^79^ Wang and co‐workers synthesized mesoporous Si/C composite, which delivered a reversible capacity of 1410 mAh g^−1^ in the first cycle and retained a capacity of 1018 mAh g^−1^ after 100 cycles at the current density of 500 mA g^−1^.^80^

Compared to solid core–shell structure, hollow core–shell structure provided additional internal void space to accommodate Si volume expansion. Yolk–shell structures have been widely investigated for further improving the performance of Si nanoparticals/carbon composites.^61^, ^81^ Cui and co‐workers have fabricated a “yolk–shell” type Si/C composite electrode,^75^ which showed excellent capacity (2833 mAh g^−1^ at 400 mA g^−1^) and cycle life. Similarly, Wang and co‐workers also fabricated yolk–shell Si@void@C nanocomposites, exhibiting considerable reversible capacities (628 mAh g^−1^ after 100 cycles) and good rate performances.^82^ To address the challenge of huge volume change and unstable SEI of silicon in cycles, Yu and co‐workers have proposed a “double‐shell” concept and prepared double carbon shells coated Si nanoparticles (DCS‐Si).^83^ The inner carbon shell provides finite inner voids to allow volume changes of Si nanoparticles inside of inner carbon shell, while the stable outer shell stabilizes the SEI layer in case of any cracks. The expansion/contraction of Si nanoparticles and diffusion paths of Li^+^ ions during charge/discharge processes in the DCS‐Si electrode. Because of this double‐shell structure, DCS‐Si electrode delivered a high rechargeable specific capacity of 1802 mAh g^−1^ at a current rate of 0.2 C, superior rate capability, and good cycling performance up to 1000 cycles. More recent designed Si‐based anodes are concluded in **Table** **2**, providing viable options for fabricating stable Si anodes for high‐energy and high‐power Li battery systems.

**Table 2:** The designed Si‐based anodes and their LIB properties

Si‐based anodes	1st and 2nd discharge capacity [mAh g^−1^]	Capacity after (*n*) cycles [mAh g^−1^]	Current density [mA g^−1^]	Ref.
Double‐walled Si@SiO*_x_* nanotube	1500	1780	≈400	58
Yolk–shell Si@C	2833	1500	≈400	75
Microparticle Si@C core–shell structure	1560	1500	≈1000	62
Hierarchical structured Si pomegranates	1270	1160	≈2100	61
Si@GO	1200	940	≈1000	63
Graphene caged Si microparticles	≈1700	1400	1500	64
3D porous Si monoxides	1520	1490	≈400	215
Stretchable graphitic carbon@Si@polymer	872	722	≈100	216
Multilayer G@Si nanoparticles	2820	–	2000	217
Hierarchical 3D mesoporous Si@G nanostructures	1480	1200	1000	218
Tremella‐like nanostructure of Si@void@G	2230	1497.3	200	219

Tin‐based anodes are an attractive anode material for next‐generation LIBs due to their high specific capacity, such as SnO_2_ (≈790 mAh g^−1^), SnO (≈875 mAh g^−1^), or Sn (≈990 mAh g^−1^). In Li/SnO_2_ anodes, the electrochemical processes can be demonstrated by the equations below^84^, ^85^

(4) $${\mathrm{SnO}}_{2}\;+\;4{\mathrm{Li}}^{+}\;+\;4e^{-}\;\to \;\mathrm{Sn}\;+\;2{\mathrm{Li}}_{2}O$$

(5) $$\mathrm{Sn}\;+\;x{\mathrm{Li}}^{+}\;+\;xe^{-}\;↔\;{\mathrm{Li}}_{x}\mathrm{Sn}\;(0\;\leq \;x\;\leq \;4.4)$$

Reaction (1) represents the largely irreversible reduction process of SnO_2_ to metallic Sn.^86^ Such an irreversible chemical transformation is partly responsible for the large initial irreversible capacity loss which is usually observed in SnO_2_‐based electrodes. Reaction (2) illustrates the reversible alloying/dealloying process between Sn and Li, which contributes to the dominant capacity of the battery cell.

Recently, it is widely acknowledged that improving the tin‐based anode materials with rational nanostructures and attaching them with carbon‐based materials are attracting paramount concern for enhancing the performance of LIBs.^87^, ^88^, ^89^ For example, 1D hollow core–shell SnO_2_/C fibers have been fabricated through coaxial electrospinning, which delivered a discharge capacity of 833 mAh g^−1^ after 500 cycles at a current density of 600 mA g^−1^. Sn@carbon nanoparticles encapsulated in bamboo‐like hollow carbon nanofibers have been prepared by pyrolysis of coaxially electrospun nanofibers, which showed a reversible capacity of 737 mAh g^−1^ after 200 cycles at a current density of 250 mA g^−1^. Inspired by those researches, a porous N‐doped carbon nanofiber selectively coupled with SnO_2_ or Sn particles via electrospinning and subsequent post‐treatments was successfully synthesized by Guo and co‐workers. Besides carbon, conductive graphene nanoribbons (GNR) are commonly applied as another carbon‐based material for hybridization with tin‐based materials. Tour et al. have prepared the GNRs/SnO_2_ composite anodes, which showed a high reversible discharge capacity of 1130 mAh g^−1^ and retain ≈825 mAh g^−1^ at the 50th cycle for current densities of 0.1 A g^−1^. In brief, thanks to incorporation with tin‐based materials, these hybrid electrodes display superior cycle and rate performance. Among different nanostructures, nanomembranes are utilized in some particular electronic and energy applications for their unique mechanical feature and special electronic structure on the surface. Taking these properties into account, the amorphous SnO_2_ nanomembranes were fabricated as anodes for LIBs and presented the outstanding performance such as long cycling life of 1000 cycles at 1600 mA g^–1^ with high reversible capacity of 854 mAh g^–1^ and high rate capability up to 40 A g^–1^.

Meanwhile, it has been demonstrated recently that germanium‐based materials (Ge and GeO*_x_*) have excellent lithium‐ion storage ability and outstanding rate performance, due to their high theoretical capacity of 1600 mAh g^−1^, fast lithium diffusivity, and high intrinsic electrical conductivity. In the light of these merits, they harvest more attentions and have been hailed as one of the most popular candidate anode materials for LIBs. In order to suppress the volume changes and increase active areas in LIBs, a series of germanium‐based materials with different nanostructures or porous structures have been designed to enhance their electrochemical performance. Furthermore, it is widely approved that carbon coating is a better approach to enhance electrical conductivity to improve their rate performance. Based on that, fully and homogeneously carbon‐encapsulated Ge and GeO*_x_* nanowires were synthesized by Yu and co‐workers, which exhibit excellent Li storage properties including high specific capacities (≈1200 and 1000 mAh g^−1^ at 0.2C for Ge/C and GeO*_x_*/C, respectively). Moreover, Guo and co‐workers have designed and synthesized unique cubic Ge@CC core–shell structures in the following process. Initially, the researchers obtained uniform GeO_2_ cubes via a simple and surfactant‐free method and then made carbon layers cover on their surfaces. After a reduction treatment, the carbon‐coated germanium oxide cubes were then completely transformed into Ge@CC. The unique cubic core–shell structure and the effective carbon coating are the effective methods to buffer the serious volume changes during lithiation/delithiation, resulting in the excellent electrochemical properties of Ge@CC.

Other group V elements are also known to be promising materials forming Li‐rich alloy systems, such as Li_3_P, Li_3_As, L_i3_Sb, and Li_3_Bi, which have high capacity values due to their low atomic packing factor in their crystal structure, leading to large accommodation of Li‐ions in the voids.^51^ Among them, element Sb is an attractive anode material, which forms alloy phases with Li such as Li_2_Sb and Li_3_Sb with a high theoretical capacity of about 660 mAh g^−1^ (Li_3_Sb). Similarly, Sb/carbon nanocomposites and Sb‐based intermetallics, especially binary compounds with other metals, have been prepared to evaluate their electrochemical performance as anodes in LIBs including Sb/carbon nanotube^90^ Sb/graphite,^91^ Sb/polyacrylonitrile,^92^ and others.^93^

### 2.3 Conversion Reaction

**Figure 4:**
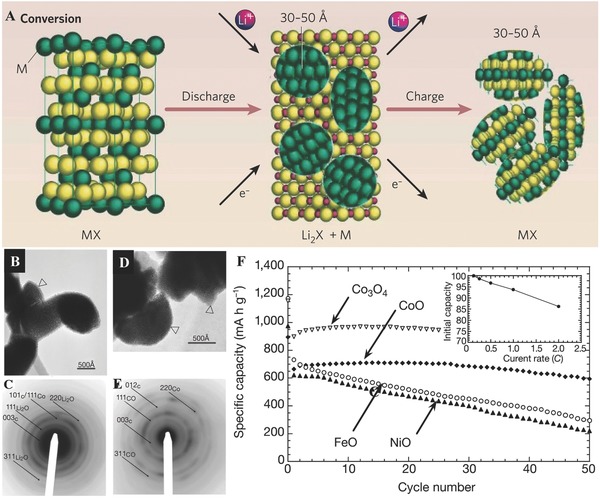
A) Schematic illustration of conversion reaction. Reproduced with permission.^95^ Copyright 2008, Nature. B) Microscopy image of the fully lithiated CoO electrode. Panels (B–F) Reproduced with permission.^97^ Copyright 2000, Nature. C) The corresponding selected‐area electron diffraction (SAED) pattern showing the presence of Li_2_O and Co inside the agglomerate. D) Microscopy image of a delithiated CoO electrode. The particle size is unaffected. E) The corresponding SAED pattern. F) The capacity of the MO/Li cells on cycling.

Among various nanomaterials, considerable research efforts have been devoted into nanostructured transition metal oxides such as iron oxide and manganese oxide due to their high theoretical capacity during the past decade. Namely, this interesting reaction between lithium and nanosized transition metal oxides is the so‐called “conversion reaction.”^94^ As shown in **Figure** **4**A, owing to the presence of nanosized transition metal particles and the stable lithium oxide, as well as Li_2_O, can be decomposed, as exemplified by the well‐known reaction nowadays:^95^, ^96^ nano‐M*_x_*O*_y_* + 2*y*Li^+^ + 2*y*e^−^ ↔ nano‐*x*M^0^ + *y*Li_2_O (M = transitionmetals; such as Co, Ni, or Fe oxide). For example, 1–2 nm metallic Co NPs dispersed in a Li_2_O matrix are observed when 100–200 nm CoO powder is fully lithiated (Figure 4B,C). The composition changes from Co to CoO during delithiation (Figure 4D,E).^97^ The capacity of the MO/Li cells on cycling showed two types of variation (Figure 4F): for FeO and NiO, the reversible capacity continuously decayed, whereas for Co oxides the capacity remained constant or even slightly increased. Most conversion‐reaction‐based transition metal oxides hold high theoretical capacities even beyond 1000 mAh g^−1^. However, there are still some drawbacks that restrict their applications in high performance LIBs. For example, the difference in voltages between the discharge and charge profiles may easily fade huge round‐trip energy density efficiency. In addition, the large volume swelling or elongation occurring during charging and discharging is bound to lead cycle fading phenomenon.^98^, ^99^, ^100^ To deal with these issues, various transition metal oxide nanostructures are commonly investigated with distinct morphologies and compositions as LIB anodes.

Iron oxide has increasingly become the dominant anode material for LIBs due to its high theoretical capacity (1007 mAh g^−1^ for Fe_2_O_3_ and 924 mAh g^−1^ for Fe_3_O_4_), low toxicity, corrosion resistance, low cost, and high availability.^101^, ^102^ Among paramount various anode materials, the α‐Fe_2_O_3_ (hematite) and Fe_3_O_4_ (magnetite) phases are the most explored iron oxides for LIB anodes.^103^ In order to release the negative effects of massive structural reorganization and volumetric changes during the conversion reaction, massive Fe_2_O_3_ nanostructures with distinct shapes and structures have been designed and measured to improve the electrochemical properties,^104^, ^105^, ^106^, ^107^, ^108^ such as nanoparticles, nanorods, and hollow nanotubes, microboxes, and microspheres. Among those nanostructures, hollow structures usually stand out for the enhanced capacity retention as shown in recent studies,^109^ due to (1) their high specific surface areas with numerous active sites for lithium redox chemistry, (2) short diffusion lengths to facilitate rapid discharge–charge rates, and (3) efficient alleviation of volume expansion during cycling.

Based on the commonly applied method to fabricating hollow Fe_2_O_3_ anode materials at present, a kind of multishelled *a*‐Fe_2_O_3_ hollow microspheres with a controlled shell thickness, porosity, and number of internal shells have been prepared by Wang and co‐workers.^110^ Different conditions about shells left a remarkable effect on the LIB performance, for example, the hollow microspheres with thin triple‐shelled demonstrating an exceptionally high stable capacity of 1702 mAh g^−1^ at a current density of 50 mA g^−1^. Mixing up with other carbonaceous materials with iron oxide is another strategy for enhancing LIBs' performance. Thanks to the existence of carbon, deformation is effectively alleviated during extensive volume expansion for carbon serving as a bracelet, and electron transfer channels are successfully built between the current collector and active materials for carbon's high conductivity. Inspired by this, Passerini and co‐workers put forward to synthesize a composite including multilayer graphene and Fe_3_O_4_. Indeed, in the light of attaching the iron oxide with carbonaceous materials, remarkable high values of specific volumetric capacity of 58.8 Ah L^−1^ and low average delithiation voltage of 0.244 V have been achieved upon applied lithiation/delithiation current of 5 A g^−1^.

Manganese oxides, as well as one kind of transition metal oxides, are appropriate for the electrode materials of LIBs,^111^, ^112^ because of the advantages of high theoretical specific capacity (such as Mn_2_O_3_ with theoretical capacity as high as 1018 mAh g^−1^), low conversion potential, low cost, and environmental benignity. Recently, Many studies have investigated the structure‐controlled synthesis of manganese oxides in order to enhance their electrochemical performances by modifying and modulating their structures.^113^, ^114^ For example, after modulating Mn_2_O_3_ microspheres from assembling porous nanosheets,^115^ they delivered a reversible capacity of 748 mAh g^−1^ at 50 mA g^−1^ over 45 cycles. α‐Mn_2_O_3_ microstructured spheres and polyhedrons also exhibited excellent lithium storage capacity of 2899 mAh g^−1^ at first cycle and 265 mAh g^−1^ after 15 cycles by structure‐controlled method.^116^ Based on morphology‐controlled decomposition of spherical MnCO_3_ precursors, porous Mn_2_O_3_ microspheres have been synthesized and showed both good rate capabilities and high specific capacities of 796 mAh g^−1^ after 50 cycles.^115^ Above all, hierarchical micro/nanostructure is regarded as an optimized design to keep the electrodes stable in charge/discharge processes in order to further improve the cycle performance of the transition metal oxide electrodes.^117^ By utilizing an improved solvent‐thermal method, Sun and co‐workers have successfully synthesized hierarchical Mn_2_O_3_ hollow microspheres that guarantee the structural stability during charge–discharge cycling and result in a reversible capacity of 580 mAh g^−1^ at 500 mA g^−1^ after 140 cycles.^118^

**Figure 5:**
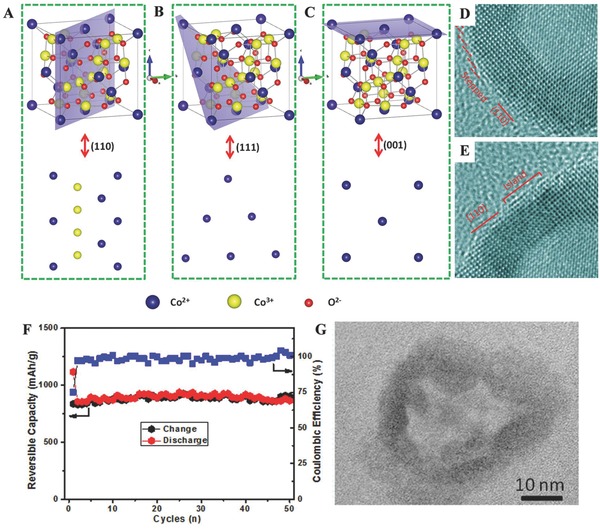
A–C) Theoretical models of the different planes of Co_3_O_4_. D,E) The edges of Co_3_O_4_ hollow nanocages. F) Cycling performance and Coulombic efficiency of CoNs at a current density of 0.2 C. G) TEM image of CoNs electrodes taken from fully discharged states after 50 cycles. Reproduced with permission.^123^ Copyright 2013, Nature.

Other transition metal oxides, such as cobalt oxide, copper oxide, nickel oxide, molybdenum oxide, zinc oxide, ruthenium oxide, chromium oxide, and tungsten oxide, have been further analyzed and developed as promising anode candidates^119^ due to their high theoretical capacity. Among them, Co_3_O_4_ nanocrystals stand out with abundant highly reactive facets that have great potential in fabricating the high‐capacity LIBs.^120^, ^121^, ^122^ Based on the systematical studies, researchers have discovered that exposed crystal plane of Co_3_O_4_ causes improvement in Li‐ion storage performance. For example, the exposed (111) planes of Co_3_O_4_ are the key factors to facilitate cyclic and rate properties comparing with nanocubes with (001) planes because of their different surface energy.^123^ Moreover, from the theoretical models of the different planes of Co_3_O_4_ (**Figure** **5**A–C), Co_3_O_4_ nanocrystals not only contain 2Co^3+^ in one‐unit cell in (110) facet, but also more Co^2+^ ions than the latter two planes (111) and (001). Hence, in principle, Co_3_O_4_ nanocrystals exhibit more excellent cycling performance and rate capabilities. When taking into account realistic factors for preparing Co_3_O_4_ nanocrystals with more exposed (110) facets, it is hard to achieve because of the high surface energy in (110) facet. Wang et al. first fabricated Co_3_O_4_ unusual single‐crystal nanocages (CoNs) with dominantly exposed (110) facets at edges of Co_3_O_4_ hollow nanocages, as can be seen in Figure 5D,E. There is a 3 nm island located on a flat (110) terrace, which is stabilized by the out‐of‐plane convex curvature of CoNs. The highly exposed (110) planes and high density of the atoms arranging as stepped on the surfaces induce the improved rate capability in LIBs. Morevoer, this unique structure of Co_3_O_4_ is also helpful to keep a good cycle performance (Figure 5F). The corrosponding TEM image in Figure 5G exhibits that the CoNs nanocages still maintain the shape integrity after 50 cycles.

### 2.4 Li Metal Reaction

**Figure 6:**
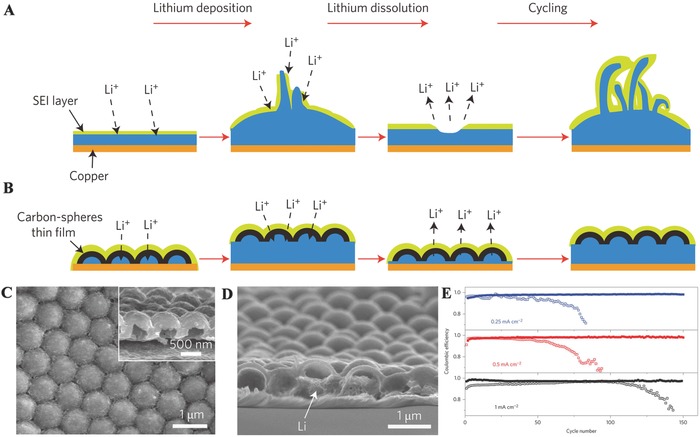
A,B) Schematic diagrams of the different Li anode structures. C) Top‐view SEM image of hollow carbon nanospheres after the initial SEI formation process. D) Cross‐sectional SEM image showing the initial deposition of Li metal under carbon nanospheres. E) Comparison of cycling performances of the hollow carbon nanosphere‐modified electrode (solid symbols) and the control Cu electrode (hollow symbols) at different current rates. Reproduced with permission.^166^ Copyright 2014, Nature.

Li metal is an attractive anode material for Li batteries,^95^, ^124^, ^125^, ^126^ owing to the high theoretical specific capacity (3860 mAh g^−1^), low density (0.534 g cm^−3^), and the lowest electrochemical potential (−3.040 V versus standard hydrogen electrode). However, the large volume changes, plating/striping of Li, and the growth of dendrites seriously restrict the application of metal lithium in commercialized Li metal batteries. During Li plating, the huge volume expansion can rupture the fragile SEI, promoting Li dendrite growth through the cracks. During Li stripping, volume contraction further fractures the SEI, while stripping from kinks in a dendrite or from its roots that can break the electrical contact between Li and the substrate. As can be seen in **Figure** **6**A, during Li deposition/dissolution cycling processes, dendritic and mossy metal deposits,^127^ formed on the surface of Li metal, can penetrate the separator and establish an internal short to the cathode, resulting in serious safety problems.^128^ Moreover, the formation of Li dendrite–electrolyte interface with high specific surface area facilitates the construction of SEI layers on the Li surface, leading to produce corrosion and isolation of Li, consequently triggering increased interfacial resistance. To overcome these problems, researchers specifically designed rational morphology of the electroplated Li for suppressing dendritic growth in order to improve cycling efficiency.

One way to reduce the irreversible volume change during cycling in Li metal is to rationally fabricate “host” (such as carbon spheres,^129^ graphene oxides,^130^ carbon^131^ and polymer nanofibers,^132^ porous Cu,^133^ and so on), because the inherent problem of Li metal is its “hostless” nature. For example, hard temples like anodic aluminum oxide membranes with small pores have been utilized as separators to keep the safety and cycle stability of Li metal.^134^ Inspired by these research, Cui and co‐workers have adopted a distinct, novel approach to suppress Li dendrites at a fundamental level via manipulating Li^+^ flux to homogeneously distribute above the anode through nanoscaled confinement, resulting in a uniform Li elongation and nucleation.^135^ The electrode is modified by coating with a thin polymer layer with vertical nanoscale channels of high aspect ratio. This strategy of spatially defined lithium growth in vertical‐aligned nanochannels provides a novel approach and a significant step toward stabilizing Li metal anodes.

There are other strategies to address the problems in Li metal by using both solid and liquid electrolytes. These protective layers are designed for controlling the formation of Li metal/electrolyte interfaces. For example, Zheng et al. constructed a coating layer of hollow carbon nanospheres that play an important role in limiting the production of lithium dendrites (Figure 6B).^136^ This hollow nanosphere structure is preserved even after cycling (Figure 6C,D) and helps isolate the lithium metal depositions and facilitates the formation of a stable solid electrolyte interphase. As shown in Figure 6E, the Coulombic efficiency is maintained at ≈99% for more than 150 cycles at 0.25 mA cm^−2^ and ≈98.5% at 0.5 mA cm^−2^. Besides carbon, boron nitride^137^ and graphene^138^ serve as chemical and physical stable barriers to restrict the reaction with Li metal. Moreover, utilization of solid‐state electrolytes (such as polymer membranes) and the electrolytes with various additives (such as vinylene carbonate, Cs^+^ and Rb^+^, LiF, lithium polysulfide, and ionic liquids) can serve as protective layers that also suppress the growth of lithium dendrites by providing an effective way for suppressing.^139^, ^140^, ^141^, ^142^, ^143^

## 3 Atomic‐Scale Studies of the Storage Mechanisms of Anode Nanomaterials

It is difficult to figure out the exact lithiation/delithiation process into LIBs, thus direct and atomistic visualization of the process can provide meaningful insights and guide the development of advanced LIBs for powering future electrical vehicles and devices.^144^, ^145^ On the basis of the above‐mentioned main mechanisms of anode nanomaterials for LIBs, more researches on observing the real‐time reaction are widely investigated. During the process, Li is stored and cycled electrochemically. A fundamental understanding of the processes that dominate the LIBs operation and electrochemical performance, especially at the atomic‐scale, is critical for the development of LIBs.^146^ Nowadays, large quantities of in situ techniques are being utilized to record the structural evolution of electrode materials during cycling (such as lithium and electron transport within the active material in electrode, electrode–electrolyte interfacial reactions, and electrolyte degradation). To investigate the lithiation/delithiation process, the commonly adopted masses of in situ techniques include: nuclear magnetic resonance (NMR) imaging,^147^ X‐ray^148^, ^149^ or Raman spectroscopic imaging,^150^ in situ atomic force microscopy (AFM),^151^ in situ TEM,^144^, ^152^ in situ spherical aberration‐corrected TEM,^153^ and 3D imaging techniques^154^ that serve as the forefront method for diagnosing the electrochemical performance of Li‐ion batteries. By combining high‐resolution characterization and electrochemical property measurements on a unique platform, it is easy for scientists to get deep insights of structure–property relationship at the nanoscale level.^155^ In this part, we will attach more attention on summarization of the in situ TEM approach, in situ X‐ray diffraction (XRD), and other atomic‐scale studies as demonstrated.

### 3.1 In Situ TEM Approach

Nowadays, in situ TEM is a powerful experimental tool that provides detailed information in operando at the atomic level during the operation of Li‐ion batteries.^156^, ^157^ In order to advance the fundamental understanding of electrochemical reactions and materials degradation, paving the way toward rational design of high‐performance LIBs is a nice shoot.^158^ It is universally believed that one of the best ways to deeply investigate some frustrations in batteries is in situ TEM battery studies, because the conventionally used liquid electrolytes in LIBs cannot be placed into the high vacuum TEM column directly.^159^ Based on that, researchers have tried to develop the imaging liquids in the electron microscope for characterizing the evolution in Li‐ion battery materials via in situ liquid electron microscopy. However, the limited spatial resolution of in situ liquid cell makes it difficult to perform real time composition analysis. There are many attempts to fabricate all‐solid electrochemical cells that exhibit good cycling performance by in situ studies, and luckily, they appear to own superb properties in the very recent experiments. Up to now, as demonstrated in the following section, the four distinct reactions have been respectively analyzed by in situ TEM.

Among numerous alloying electrode materials, Si possessing high theoretical capacity with ≈4200 mAh g^−1^ attracts considerable research interests. In order to reveal the reason for capacity fading, in situ characterization technologies were implemented to characterize the evolution processes of Si. The lithiation processes of Si follow the process: from crystalline Si to amorphous Li*_x_*Si, then crystalline Li_15_Si_4_. But the volume expansion of Si nanowires during lithiation process was anisotropic, which is determined by the orientation dependence of interfacial mobility.

**Figure 7:**
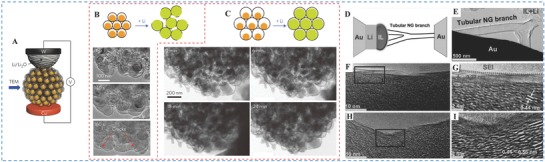
A) Schematic of the in situ TEM device. Panels (A–C) Reproduced with permission.^61^ Copyright 2016, Wiley. In situ TEM of the lithiation of silicon pomegranates with B) insufficient void space and C) sufficient void space. D) Schematic illustration of a tubular trident prototype LIB device under in situ TEM. Panels (D–I) Reproduced with permission.^20^ Copyright 2014, Nature. E) TEM image of a microscopic LIB. F–I) In situ TEM images of tube during intercalation/deintercalation process.

As mentioned above, the hierarchical silicon was fabricated as anode material by Cui and co‐workers.^61^ **Figure** **7**A schematically shows the in situ TEM set‐up. Compared with the insufficient (≈15 nm) gap size (Figure 7B), it is clear to say that under the sufficient gap size (Figure 7C) silicon expands inside the carbon framework and then occupies the void spaces, resulting in little change to both the carbon shell and secondary particle size. As can be proved by these results, it indicates that in situ TEM is a powerful and reliable way to study properties of nanomaterials at atomic scale, especially in field of the energy storage. As for the intercalation/deintercalation process, Wang and co‐workers have fabricated the branched N‐doped graphitic (BNG) tubular foam and then investigated the mechanism by in situ HRTEM. Thanks to the in situ HRTEM, they have the opportunities to directly visualize the Li^+^ storage mechanism during a lithiation process in real time, which devotes more into figuring out the storage mechanism. As shown in Figure 7D, an in situ battery prototype was constructed to conduct experiment in real time.^20^ From the TEM images (Figure 7E–I), it is obvious that the formation of SEI film, the lattice spacing, and the thickness of the SEI are the valuable factors to unveil the contributions from both curved and flat portions in a BNG branch. The structural changes in lithiation based on alloying/dealloying reactions can also be clearly demonstrated via in situ TEM study.^98^, ^160^

The mechanism of conversion‐type anodes for LIBs can also be analyzed by applying in situ TEM technique.^161^, ^162^, ^163^, ^164^ Conversion mechanism is another electrochemical working principle based on the reversible formation–decomposition of a Li_2_O nanomatrix within transition metal oxide anodes. The capacities reported are high (500–1500 mAhg^−1^), but many issues (such as huge irreversible capacity loss in the first cycle, large volume variation, higher redox potential, polarization, and poor cycle‐ability) have hindered the commercialization of these anodes. Hence, it is urgent to deeply understand the mechanisms governing the battery performance through in situ technique, and to optimize the regarded LIBs' anodes.

**Figure 8:**
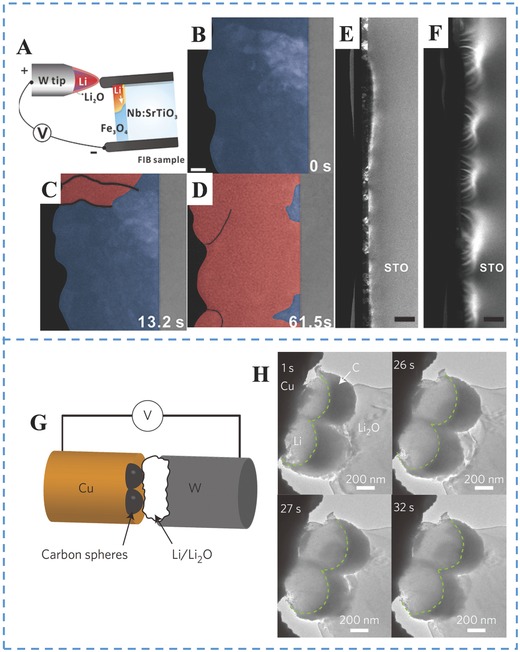
A) Schematic illustration of a dry electrochemical cell inside a TEM. Panels (A–F) Reproduced with permission.^165^ Copyright 2017, Wiley. B–D) ADF‐STEM image series were captured in real time. E) Before and F) after the in situ lithiation. G) Schematic showing the configuration of the in situ TEM cell. H) In situ TEM of the Li deposition process on Cu wires decorated with hollow carbon nanospheres taken at different times. Panels (G,H) Reproduced with permission.^166^ Copyright 2014, Nature.

Su and co‐workers have constructed a Fe_3_O_4_ thin film battery to investigate the properties in LIBs combined with in situ TEM technique.^165^ **Figure** **8**A presents a schematic of the open cell configuration. The direct observation in real‐time of the images in Figure 8B–D illustrates the conversion process in the red‐colored area. The conversion reaction starts accompanied by volume expansion, resulting in the filled‐n racks. To focus on preexisting epitaxial strain that can be used to control the electrochemical reaction in thin film battery, the comparison images before and after the lithiation in Figure 8E,F are to exhibit that the lithiation is suppressed by the compressive interfacial strain. For instance, the mechanism in Li metal anodes is provided by Cui and co‐workers that have constructed the Li metal anode coating with a monolayer of interconnected amorphous hollow carbon nanospheres and then carried out in situ TEM experiments to uncover the Li deposition phenomenon within the hollow carbon nanospheres.^166^ Figure 8G is on behalf of the schematic illustration of cell. A thin layer of Li*_x_*O formed on the Li metal (Figure 8H) to act as a solid electrolyte for the nanoscale electrochemical cell. Li first deposited on the Cu wire underneath the carbon nanospheres immediately after application of the voltage bias is recorded and attested by a series of bright‐field TEM images of the carbon nanospheres during the Li deposition process in Figure 8H. With the Li continuous deposition, the average thickness of the Li is increased in the carbon nanospheres, which confirms that depositing Li underneath carbon is hard for maintaining the integrity of the carbon nanospheres.

### 3.2 In Situ XRD Approach

The in situ XRD for battery research has been widely adopted to monitor the phase transitions of the electrodes during lithiation/delithiation. The cell architecture for the XRD measurements incorporates an X‐ray transparent window, which allows for the X‐rays to reach the electrodes during operations. Indeed, in recent decades, many reports on in situ XRD measurements of battery electrodes have been published.^167^, ^168^, ^169^, ^170^, ^171^ Several types of cells were developed in which a Li‐insertion electrode is measured exclusively, either in the transmittance or reflectance mode, in a way that maximizes the signal‐to‐noise ratio. A very high resolution response can be obtained using an X‐ray beam from a synchrotron source,^172^ which subsequently increases to obtain fast measurements of operando half cells. In those studies, the cells were operated in transmission mode and thus require an additional X‐ray window.

**Figure 9:**
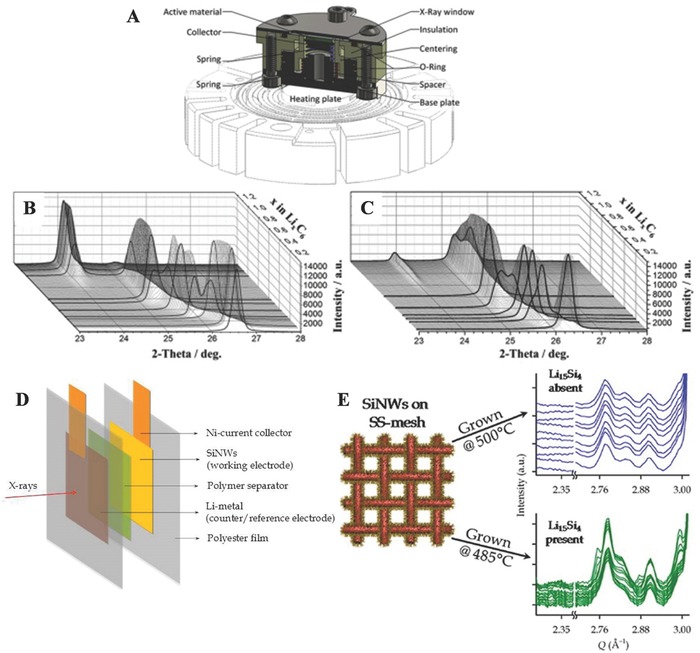
A) Schematic of in situ XRD‐cell for measurements at room and elevated temperature. Panels (A–C) Reproduced with permission.^173^ Copyright 2017, Elsevier B.V. X‐ray diffractograms of operando measurements on a Li/graphite cell at B) 30 °C and C) 47 °C. D) Schematic figure of an in situ cell. Panels (D,E) Reproduced with permission.^21^ Copyright 2012, American Chemical Society. E) In situ XRD results for a SS‐mesh cell cycled at C/5 in different temperature.

As shown in **Figure** **9**A, an in situ XRD cell is demonstrated and used by Cañas et al. for first time to present an operando XRD analysis of a battery at elevated temperatures.^173^ The in situ cell was developed to simultaneously investigate the structural and electrochemical behaviors of batteries at room and elevated temperatures. In their study, a graphite anode was used to validate the in situ cell design, and the intercalation and deintercalation processes during battery cycling were investigated at 30 and 47 °C (Figure 9B,C). Contrary to the well‐known sequential process of lithium intercalation into graphite at room temperature, at elevated temperature, the sequence was different, and the simultaneous appearance of up to four phases was observed during the intercalation and deintercalation processes, which would be of great interest for real battery applications.

Misra et al. use an X‐ray transparent battery cell (Figure 9D) to perform in situ synchrotron X‐ray diffraction on Si nanowires in real time during electrochemical cycling.^174^ They found that the presence of this crystalline phase degrades cycling performance compared to Si nanowires, where no intermediate crystalline phases are formed, and the formation of this crystalline phase is correlated with the Si nanowires growth temperature (Figure 9E). Su and co‐workers extended the in situ XRD studies to investigate the lithiation/delithiation mechanism in TiO_2_ hollow spheres.^175^ To futhure investigate the formation and component of the discharge products, in situ high‐energy X‐ray diffraction was employed to monitor the evolution of a lithium–oxygen battery in discharge processes, taking RuO_2_/MnO_2_ as the anode.^176^ The research indicated that the formation of Li_2_O_2_ had occurred during the discharge process, thus resulting in fast capacity fading. Therefore, the in situ XRD analysis results provided extremely insightful evidence for the mechanism and kinetics studies in LIBs and will open new strategies of designing electrode materials for high energy density LIBs.

### 3.3 Other In Situ Techniques

Apart from the in situ TEM, some other in situ techniques are applied to probe the interfacial effects and volatile products when LIBs are in operando. For example, spherical aberration‐corrected STEM,^177^ cryo‐EM, 3D imaging techniques,^72^ neutron reflectometry,^178^, ^179^ NMR spectroscopy,^180^, ^181^, ^182^ AFM,^183^, ^184^, ^185^ Raman spectroscopy,^186^, ^187^ and X‐ray absorption spectroscopy^188^ have been widely applied for these in situ studies. In this section, we will focus on the spherical aberration‐corrected STEM, cryoelectron microscopy, and 3D imaging techniques.

#### 3.3.1 Spherical Aberration‐Corrected Electron Microscope Approach

Significant progress displayed by atomic imaging of light elements has been probed by using advanced microscopy techniques.^189^, ^190^, ^191^ In particular, spherical aberration‐corrected STEM can not only benefit from mapping oxygen defects, but also from detecting the light elements Li.^192^ Therefore, the spherical aberration‐corrected STEM technique in combination with first‐principles calculations is proved to have great power in the energy storage of anode at atomic level.^193^, ^194^ For example, more clear and direct visualization of Li ions in LiCoO_2_ was achieved by spherical aberration‐corrected STEM technique than using high‐resolution TEM.^153^ Owing to the spatial resolution of spherical aberration‐corrected STEM technique is about ≈0.40 Å, it has been proved to be a powerful tool in determining the fine structure in Li‐ion battery system, because the researches about visualizing the Li storage site (Li radius: 0.68 Å) and investigating the local structure and surface/interface structures are conducted and testified the capability of the bulk probing techniques.

**Figure 10:**
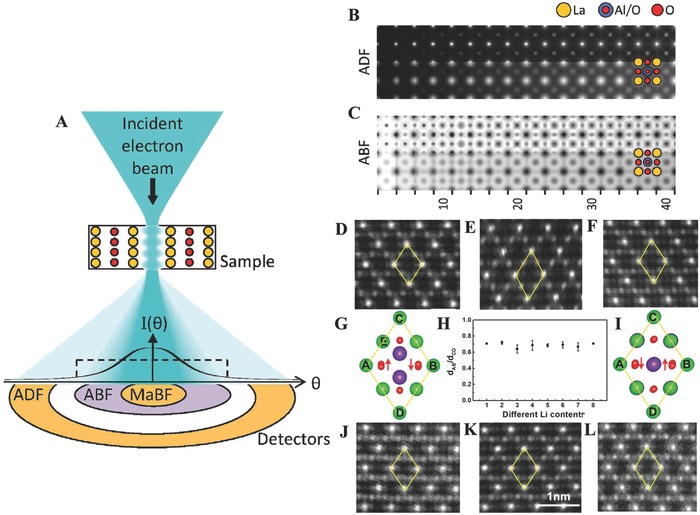
A) Schematic of some key detector geometries in atomic resolution spherical aberration‐corrected STEM. Panels (A–C) Reproduced with permission.^198^ Copyright 2014, Elsevier B.V. B) ADF and C) ABF images as a function of thickness for LaAlO_3_. D–L) Spherical aberration‐corrected STEM‐HAADF images and structure illustrations of Li_4_Ti_5_O_12_ samples with different Li contents at [110] zone axis. Panels (D–L) Reproduced with permission.^153^ Copyright 2015, American Chemical Society.

For atomic resolution structure analysis, annular dark field (ADF) imaging in spherical aberration‐corrected STEM has long provided robust and directly interpretable images, but the thermal‐scattering‐dominated contrast strongly favors heavy elements.^195^ ADF has recently been complemented by the development of annular bright field (ABF) imaging,^196^, ^197^ involving an annular detector in the outer area of the bright field (forward scattering region) and enabling the simultaneous imaging of light and heavy elements with similar contrast. **Figure** **10**A shows the schematic of the electron probe “channeling” (scattering) along a column of atoms and the resultant intensity profile in the diffraction pattern as a function of angle θ away from the optical axis. Here is an example of ADF and ABF images of LaAlO_3_ viewed along the [001] direction, as a function of specimen thickness is compared in Figure 10B,D.^198^ As expected, the ADF images in Figure 10B are dominated by the heavy La column, with the Al/O column faintly visible but the O columns invisible. The ABF images assuming perfect partial coherence, the top half of Figure 10C, show all columns—La, Al/O, and pure O—consistent with the previously touted utility of ABF for imaging light elements.

In corporation with spherical aberration‐corrected STEM techniques, Demopoulos and co‐workers employed Li_4_Ti_5_O_12_ spheres as Li‐ion battery anode and carefully investigated their bulk/surface structure changes during lithiation at atomic‐scale.^153^ Without doubts, the spherical aberration‐corrected STEM‐high‐angle annular dark field (HAADF) technique is an effective way to image the Ti columns and the Li_4_Ti_5_O_12_ (LTO) spinel framework during lithiation. As shown in Figure 10D–L, the HAADF images demonstrate that a series of LTO structures with different Li contents are detected at [110] zone axis. As can be seen in Figure 10, the schematic repeated units of Li_4_Ti_5_O_12_ without/with lithiation are denoted by the yellow quadrilateral ACBD (Figure 10D,F). By the way, the ratio of *d*_AB_/*d*_CD_ is used to record the LTO nanostructure evolution and eliminate errors by the spherical aberration‐corrected STEM itself. Therefore, there is no doubt to provide an effective way to easily detect the subtle structure changes upon lithiation/delithiation. When Li_4_Ti_5_O_12_ is fully lithiated with the Ti 3d orbitals partially occupied, the Ti ions are reduced from Ti^4+^ to Ti^3.4+^. While, with the help of spatial atomic‐resolution electron energy‐loss spectroscopy, it is found that inhomogeneously distributed electrons are also found in lithiated Li_4_Ti_5_O_12_, i.e., Ti ions are in different valence states according to their local environment. The calculations indicate strong coupling between the charge state of Ti and the distribution of Li, supporting the hypothesis of a strong Li^+^–e^−^.

#### 3.3.2 Cryoelectron Microscopy

**Figure 11:**
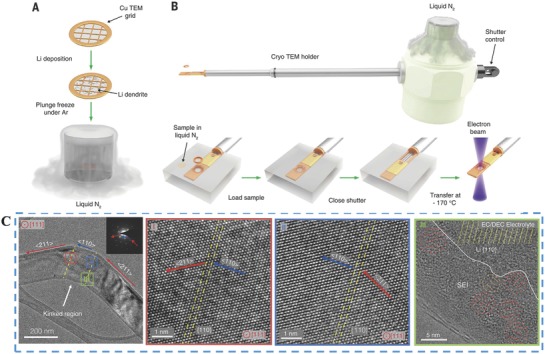
A) Li metal dendrites are electrochemically deposited directly onto a Cu TEM grid and then plunged into liquid nitrogen after battery disassembly. Panels (A–C) Reproduced with permission.^200^ Copyright 2017, Science. B) The specimen is then placed onto the cryo‐TEM holder while still immersed in liquid nitrogen and isolated from the environment by a closed shutter. C) Atomic‐resolution TEM of kinked Li metal dendrite and SEI interface.

As we know, elucidating the Li metal and SEI nanostructure is critical for developing high‐performance LIBs. TEM is one of the most common ways to study battery materials. However, Li metal is very reactive at room temperature and corrodes upon brief air exposure during sample transfer into the TEM column. Additionally, a low melting point and weak atomic bonding make the light Li atoms extremely unstable under an electron beam.^199^ To overcome these challenges, Cui and co‐workers have developed a cryotransfer method (**Figure** **11**A,B) based on cryo‐EM procedures used in structural biology.^200^ At cryogenic temperatures, Li metal does not react with the liquid nitrogen or ice so that the dendrites retain their electrochemical state with the relevant structural and chemical information preserved. Atomic‐resolution TEM of kinked Li metal dendrite and SEI interface are clearly shown in Figure 11C. This work presents a simple methodology to preserve and image sensitive battery materials with atomic resolution, revealing detailed nanostructures. The insight gained from these experiments can lead to a more complete understanding of the failure mechanisms in high‐energy batteries.

#### 3.3.3 3D Imaging Techniques

Based on recent studies, 2D imaging is sufficient for identifying density changes in electrode materials and observing fracturing and pulverization of particles for their large volume changes. However, to quantify volume changes and verify the complete fracturing of a particle into multiple pieces, one must know 3D imaging with high precision.^201^, ^202^ X‐ray crystallography as the primary method has been utilized to solve the 3D atomic structure of crystals in the last century.^203^ Nowadays, the modern technologies have been developed to determine the 3D atomic structure of crystal defects such as grain boundaries, stacking faults, dislocations, and point defects, as well as to precisely localize the 3D coordinates of individual atoms in materials without assuming crystallinity.^204^, ^205^, ^206^, ^207^ For example, based on the 2D image from ADF‐STEM, the sample can be rotated around a tilt axis and a series of 2D images will be measured at different tilt angles to acquire a tomographic tilt series. After this, the coordinates of individual atoms are calculated, and then traced and refined to produce the 3D atomic model of the sample.^208^

**Figure 12:**
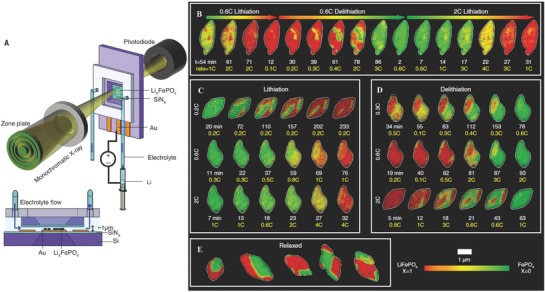
A) Schematic of the operando liquid imaging platform. B–D) Operando Li composition frames of different particles. E) Ex situ frames of Li composition for relaxed particles. Reproduced with permission.^208^ Copyright 2016, Science.

Lim et al. have setup an operando X‐ray microscopy platform to probe 3D maps of a typical particle undergoing multiple delithiation and lithiation cycles (**Figure** **12**A).^208^ As shown in Figure 12B–E, it has been tracked that several particles (de)lithiate at 0.2, 0.3, 0.6, and 2 C. With the high electrochemical fidelity, those operando Li composition maps exhibit distinctively in composition across each particle, which can share more efforts to confirm that there are increasing global current increases in the rate of (de)lithiation of individual particles. Above all, phase separation under the influence of elastic strain can dominate the equilibrium Li distribution from the ex situ frames of Li composition for relaxed particle in this study.

## 4 Conclusion and Perspectives

**Figure 13:**
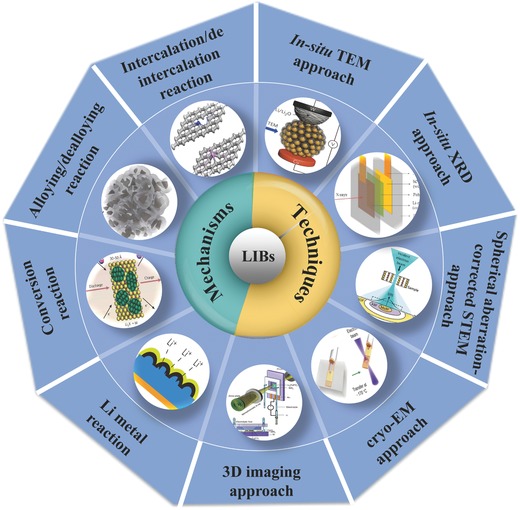
Schematic of the research areas for high capacity LIBs. Reproduced with permission.^21^ Copyright 2017, RSC;^72^ Copyright 2017, Science;^95^ Copyright 2008, Nature;^166^ Copyright 2014, Nature;^61^ Copyright 2016, Wiley;^21^ Copyright 2012, ACS;^198^ Copyright 2014, Elsevier B.V.;^200^ Copyright 2017, Science;^208^ Copyright 2016, Science.

Continuous progress in LIBs has been witnessed due to the development of high‐capacity anode materials recently. In order to discover alternative anodes materials beyond graphite, various multielectron anode materials have been explored in research, such as Si, Sn, Ge, Li, and so on. Those new developed anodes may work on different principles and fields, such as intercalation/deintercalation reaction, alloying/dealloying reactions, conversion reaction, and Li metal reaction. In this review, we first summarize the recent advances in anodes in each reaction, then we conclude the atomic‐scale studies of the storage mechanism in LIBs (**Figure** **13**), which are based on a direct observation of the lithium process and the structure–performance relationships.

Even though much important progress has been achieved in the high‐capacity anodes in LIBs these days, the reaction mechanism of some electrode materials is still unclear and some problems associated with the mechanism analysis have not been answered as yet. This area still faces many challenges that need to be addressed:

(1) It is urgent to develop new cutting‐edge microscopic methods for rechargeable battery mechanism studies. For example, seeing light elements in electrode materials under atomic resolution (such as ordered Li and O vacancies) would provide essential information for the special Li^+^ ion transport paths. Moreover, control of the interfaces between electrode and electrolyte is another key factor to determine battery performance. However, it is still challenging to visualize Li^+^ diffusion across the interface employing the current TEM techniques.
(2) There is still a big gap between the nanobatteries and the real batteries. As we known, the crucial task for in situ characterization is the smart construction of an in situ cell. However, some of the present in situ cells are incapable of completely mimicking real battery‐operation conditions. For example, the formation of SEI layers can be observed through in situ liquid TEM, but the normally used electrolyte can barely be applied at such conditions. Other in situ TEM cells have been fabricated into a nanobattery configuration using a Li_2_O layer as a solid electrolyte, but the surface/interface information can also not be accurately obtained for such systems.
(3) It is highly desirable to expand the in situ technique to dynamic analysis. Current in situ techniques are focused on static studies, while less attention is paid to the kinetics. While, the battery reaction feasibility is associated with dynamic properties. Thus, the nonequilibrium states should be probed, which requires a fast data‐collection rate and is not possible using most of the present tools.
(4) Theoretical calculations are necessary and important for deeply analyzing and understanding the storage mechanisms peculiar to electrodes. These will guide searching and designing better anode electrode materials. A combination of theoretical calculations and in situ observations of cation transport can uncover the transport/storage mechanisms of Li^+^ ions inside new anode electrode nanomaterials during electrochemical cycles.
(5) Introducing external stimuli for enhanced perforamence of LIBs is expected, even if the actual mechanisms are still in question and their optimization pathways are still unclear. For example, the introduction of light into rechargeable batteries can improve the charge processes and complete charging at a lower potential. Moreover, a suitable magnetic field can also drive a concentration cell under the assistance of magnetic particles.
(6) The profound insights into the novel energy storage mechanisms are urgent to be entirely disclosed. With the help of new preparation approaches and advanced microscopic and novel calculation methods, more and more nanostructured materials and their hybrids should be designed and fabricated. It is conceivable that these novel mechanisms will definitely guide a smart design of new electrode materials and thus greatly promote the rapid development of new energy storage materials and technologies, which are environmentally benign and cheap.

Hence, there is still a long way to go for the study of this field. The fabrication and modification of anode materials with more excellent electrochemical properties attract more efforts to devote. Meanwhile, increasing fundamental researches will be carried out to investigate the structure–property relationships in anodes. Third, it is anticipated that the new but rapidly emerging field of in situ electrochemical techniques will continue to bring exciting new fundamental understanding of battery reaction mechanisms and their fading routes. Certainly, it should also extend the development of smart in situ cells to other powerful analytical equipment.

## Conflict of Interest

The authors declare no conflict of interest.
